# Improving Recruitment Into Research Studies via Electronically Collected Patient-Entered Data: Mixed Methods Study

**DOI:** 10.2196/77720

**Published:** 2025-10-29

**Authors:** Irene Katzan, WH Wilson Tang, Andrew Schuster, Ryan Honomichl, Misti Allison, Renee Feldman, Michelle Gandolf, Benjamin Walter, Brittany Lapin

**Affiliations:** 1 Center for Outcomes Research & Evaluation Neurological Institute Cleveland Clinic Cleveland, OH United States; 2 Heart Vascular and Thoracic Institute Cleveland Clinic Cleveland, OH United States; 3 Market Research and Analytics Cleveland Clinic Cleveland, OH United States; 4 Lieberman Research, Inc Great Neck, NY United States; 5 Center for Neurological Restoration Neurological Institute Cleveland Clinic Cleveland, OH United States; 6 Quantitative Health Sciences Cleveland Clinic Cleveland, OH United States

**Keywords:** patient recruitment, patient-reported outcomes, qualitative evaluation, survey, clinical research

## Abstract

**Background:**

Patient recruitment remains a critical challenge in clinical research. Although the integration of electronically collected patient-entered data within clinical practices enables innovative recruitment approaches, existing methods present challenges such as increased patient burden and potential violation of autonomy. A more nuanced approach involves identifying patient attributes associated with higher propensity for research participation, enabling research teams to efficiently prioritize outreach efforts.

**Objective:**

This study aims to (1) develop patient-reported questions reflecting perceptions about research participation and (2) determine whether patient responses are predictive of interest in joining a precision medicine registry.

**Methods:**

This mixed methods study used an exploratory sequential design in 2 phases. Phase 1 involved cognitive interviews with 32 patients recruited through the Cleveland Clinic Healthcare Partners program to develop “research perception” questions. Participants evaluated 9 candidate questions that were based on a literature review of research participation factors. Three questions were selected for implementation. Phase 2 was a cross-sectional cohort study incorporating these 3 questions into routine electronic questionnaires completed by primary care patients through the patient portal. The study population included 1077 patients who completed both “research perception” and “research recruitment” questions between August 2018 and April 2019. Diagnostic accuracy was assessed using receiver operating characteristic curve analysis, and multivariable logistic regression models evaluated associations while adjusting for demographic and health factors.

**Results:**

Phase 1 revealed strong research support among participants, with 97% (31/32) agreeing that research should be part of the institution’s mission and 100% (32/32) affirming that research enhances patient care. Phase 2 included 1077 patients (mean age 48.3, SD 16.3 years; 625/1065 female, 58.68%; 661/1005 White, 65.77%), of whom 278 (25.8%) expressed interest in being contacted about the precision medicine registry. Patients expressing interest were older and had worse self-reported health, more depressive symptoms, and greater social needs. “Strongly agree” and “very important” responses to any “research perception” question were significantly associated with study interest, with adjusted odds ratios ranging from 6.36 (95% CI 2.77-14.6) to 17.6 (95% CI 5.08-61.1; *P*<.001). The “research perception” questions demonstrated high sensitivity (>80%) but limited specificity (24%-31%).

**Conclusions:**

Patient-reported questions assessing research participation likelihood can help identify patients more likely to enroll in clinical studies. This approach enables effective recruitment prioritization while preserving patient autonomy and reducing patient burden. High sensitivity makes these questions valuable as screening tools, although limited specificity suggests use for prioritizing rather than excluding participants. Further validation across different trial types and populations is warranted.

## Introduction

### Background

Recruitment of patients into clinical research studies is a persistent and well-documented challenge [1]. The number of clinical trials open for patient enrollment has increased dramatically over time, growing from 4000 protocols in 2000 to more than 134,000 in 2023, which has intensified the challenge of patient recruitment [2]. Approximately 80% of clinical trials are delayed or terminated early because of recruitment problems [3]. Poor patient recruitment is the top cause of clinical trial delays [4]. Failure to meet recruitment goals delays treatment advances, threatens internal validity, and raises concerns about the generalizability of results [3]. There is a clear need to improve recruitment practices.

The integration of electronically collected patient-entered data within clinical practices offers innovative approaches to aid research recruitment [5,6]. While patient-entered data can help identify potential study participants through eligibility screening and interest assessment, these methods present distinct challenges. Using electronic patient questionnaires to screen for study eligibility, while seemingly straightforward, increases the burden of patient response and can create frustration among patients uninterested in research participation [7]. This approach also requires ongoing technical resources to maintain and update screening criteria as studies evolve. More problematically, additional recruitment outreach to patients who have declined participation via questionnaire violates patient autonomy—a cornerstone of ethical research [7]. This practice risks eroding trust in the health care system.

A more nuanced approach involves identifying a patient attribute associated with a higher propensity for research participation. This strategy enables research teams to prioritize outreach efforts efficiently to those with a higher likelihood of participating while avoiding prescribed eligibility criteria or direct elicitation of their interest in research. This method reduces the risk of alienating patients and reduces patient burden of questionnaire completion.

### Objectives

The objectives of this study were to (1) identify patient-reported questions that reflect perceptions about participating in clinical research studies and (2) determine whether patient responses to these questions are predictive of interest in participating in a precision medicine research registry.

## Methods

### Overview

**Figure 1:**
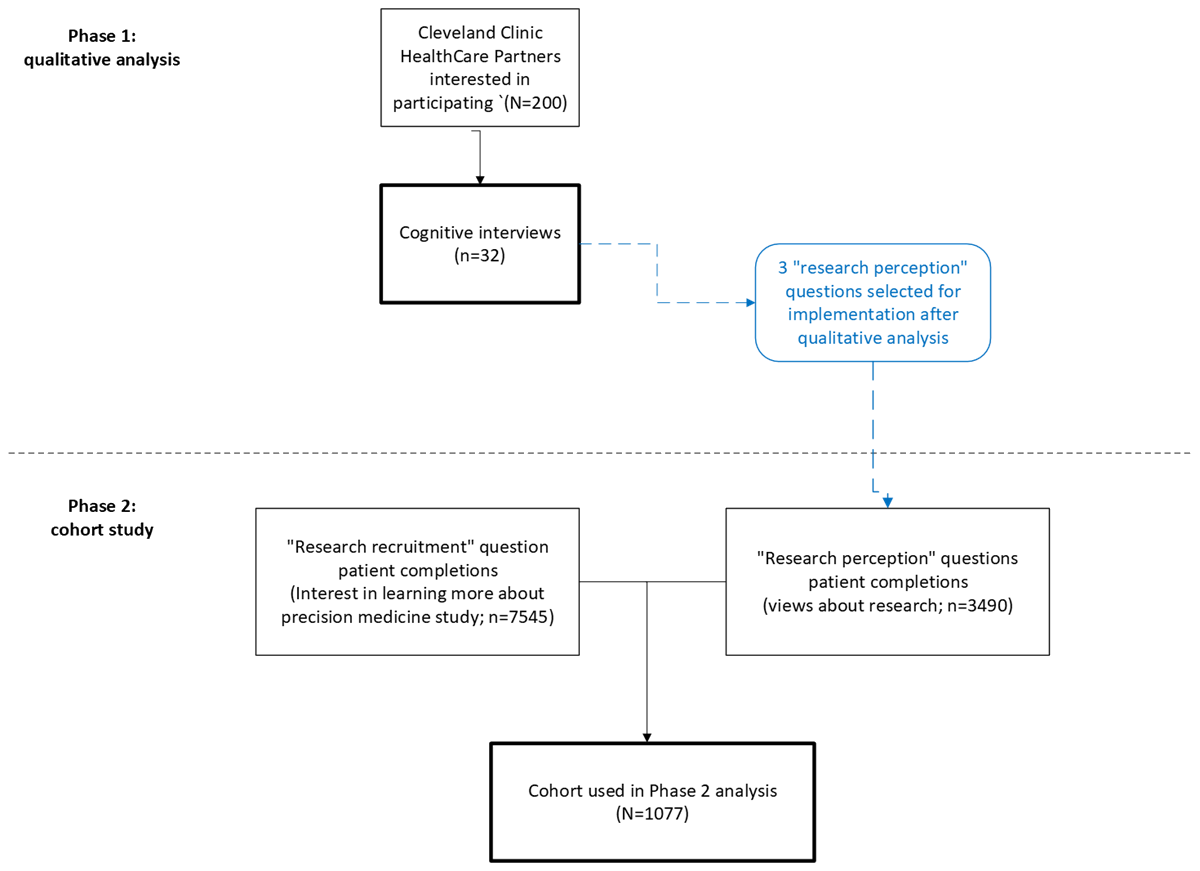
Study workflow. *Cleveland Clinic health care partners are patients who volunteer to provide their perspective about the design and delivery of care.

This was a mixed methods study that used an exploratory sequential design. It was conducted in 2 phases. Phase 1 was a qualitative analysis that informed the phase 2 quantitative study. In phase 1, cognitive interviews were conducted to identify attribute questions that would reflect the likelihood that patients would participate in a clinical research study. In phase 2, the relationship between patients’ responses to the questions, identified in phase 1, and their interest in learning more about an ongoing research study was assessed in patients seen in primary care clinics. The identified “research perception” questions were added to an electronic questionnaire set that patients routinely complete before primary care office visits. The set also included a question about interest in being contacted about an ongoing precision medicine registry (Figure 1).

### Ethical Considerations

Phase 1 of this study was reviewed by the Cleveland Clinic Institutional Review Board and considered preparatory for research. Participants in phase 1 received an information sheet inviting them to participate in the cognitive interviews, which outlined the study’s purpose, participation requirements, and voluntary nature of their involvement. Each participant received a US $25 Amazon gift card as compensation. Phase 2 received exemption from the Cleveland Clinic Institutional Review Board (IRB 19-465), which determined that informed consent was not required for either phase of the study. Project data were securely stored and analyzed on Cleveland Clinic servers, with access restricted to authorized study personnel.

### Phase 1: Qualitative Study to Identify Optimal Question on Perception of Research

#### Overview

Patients were recruited through the Cleveland Clinic Healthcare Partners program, which is comprised of patients who volunteer to provide their perspective about the design and delivery of care. Of the 4590 Healthcare Partners members, 1000 (21.79%) were randomly selected and sent an online invitation to participate in a cognitive interview. Of these 1000 patient panel members, 200 (20%) indicated interest in participating. Of these 200 potential participants, 32 (16%) were selected using purposive sampling to obtain a representation of patients across levels of patient-reported health status, sex, age, and race.

#### Cognitive Interviews

After informed consent, 30-minute patient interviews were conducted via videoconference calls between October 31, 2017, and November 14, 2017, to develop self-reported questions that would reflect patients’ likelihood of participating in a clinical research study. All sessions were conducted by an experienced qualitative researcher (RF) and audio recorded and transcribed.

The specific aims of the interviews were to (1) explore the factors that impact the decision to participate in clinical research; (2) evaluate the clarity of, and participant responses to, 9 potential questions; and (3) identify alternative questions that may reflect patients’ likelihood of participating in a research study that were not previously considered. The 9 potential questions were selected based on a literature review of factors considered important in patients’ decisions to participate in clinical research [8-20] as well as the feasibility of assessment through patient questionnaires. An interview guide was developed (Multimedia Appendix 1) that contained candidate “research perception” questions. Some of the candidate “research perception” questions focused on participants’ overall impression of research, while 2 questions asked about the relevance of potential benefit to the patient versus benefit to others. Several questions asked about different factors that may affect their decision to participate, such as risk, location, and time commitment. Finally, there was a question about the importance of being informed about ongoing research trials for which they would be eligible. Participants were asked to respond to each of the candidate “research perception” questions on a 4-point Likert scale. They were then asked to explain the reasons for their responses.

#### Qualitative Analysis

The study used a deductive and explanatory approach to qualitative analysis. This approach was chosen because it provided a more structured investigation, allowing for a comprehensive understanding of patients’ perspectives on the best question to assess their interest in participating in potential research projects [21]. Field notes were recorded for each interview and were supplemented by transcripts for verbatim quotes and details. Two qualitative analysts then coded the data for themes and subthemes under the supervision of the senior qualitative researcher (RF). The coding tree was organized into 4 overarching themes that were identified before the start of interviews as part of the research objectives: level of interest to participate and past participation in clinical research at Cleveland Clinic, general perceptions of the importance of clinical research, drivers to participate in clinical research, and areas for improvement to recruit appropriate patients into clinical research. These themes reflect attitudinal motivations that drive patients to participate in clinical research. Subthemes were developed to understand the more nuanced aspects of the data, including the varying drivers for participating in clinical research and recommendations to improve the patient recruitment process. The hierarchical structure of the content analysis was organized from general categories to specific topics. A summary report was developed that included findings with recommendations.

### Phase 2: Cross-Sectional Cohort Study to Evaluate Questions From Phase 1

#### Overview

Phase 2 was a cross-sectional cohort study to evaluate whether the “research perception” questions identified in phase 1 could predict patient interest in an ongoing precision medicine research registry. From November 13, 2018, to April 19, 2019, the “research perception” questions from phase 1 were added to the standard patient questionnaires that patients routinely completed through the electronic health record (EHR) patient portal (MyChart [Epic Systems Corporation]) before their primary care visits. As part of the standard of care, patients complete these electronic questionnaires either through the patient portal before arrival or by using tablets and computer workstations after arrival. Patients may decline to complete questionnaires or skip individual questions, and responses are immediately available to health care providers within the EHR.

From May 9, 2017, to April 19, 2019, all patients at select primary care locations also answered a “research recruitment” question asking whether they were interested in being contacted to learn more about a precision medicine registry (Multimedia Appendix 2). They were asked this question only once. Those who expressed interest selected their preferred contact method (telephone or email) and were subsequently contacted by a research coordinator through a separate process unrelated to this study.

The phase 2 patient population included primary care patients who completed both the “research perception” and “research recruitment” questions during primary care visits between November 13, 2018, and April 19, 2019.

#### Study Variables

In addition to responding to the “research perception” and “research recruitment” questions, patients completed the Patient-Reported Outcomes Measurement Information System Global Health (PROMIS GH) scale, the Patient Health Questionnaire-2 (PHQ-2) depression screen, and internally developed social needs questions. The PROMIS GH is a widely used, generic health measure comprising 10 items that generate mental and physical health summary scores, with higher scores indicating better health status [22]. The PHQ-2 includes 2 items, with higher scores reflecting more severe depressive symptoms [23]. Scores above 3 are indicative of at least moderate depressive symptoms. The social needs questions had binary (yes or no) responses and asked the following: (1) “In the last 12 months, has it been hard for you to pay any of these bills?” (2) “In the last 12 months, have you had to forgo healthcare because you didn’t have a way to get there?” (3) “In the last 3 months, were you ever worried your food would run out before you could buy more?” (4) “In the last 12 months, did you ever sleep in a shelter or not have a steady place to live?”

Additional demographic variables extracted from the EHR included age, sex, race, marital status, and median household income estimated from the 2010 census using zip codes. The authors had full access to the data from the study population.

#### Analysis

Descriptive statistics were used to evaluate responses to the “research perception” questions and to compare the characteristics of patients according to their responses to the precision medicine “research recruitment” question. Characteristics were compared by response to the precision medicine “research recruitment” question using the 2-tailed *t* test for continuous variables and the chi-square test for categorical variables.

Multivariable logistic regression models were constructed to predict response to the “research recruitment” question. Each of the 3 “research perception” questions were included in separate models to evaluate the ability of these questions to independently predict patients’ interest in being contacted to learn more about the ongoing research study if combined with other clinical variables available in the EHR. The independent variable in these models was one of the “research perception” questions. The dependent variable was patient response to the “research recruitment” question. Covariates included age, sex, race, marital status, median household income, PROMIS GH physical and mental health summary scores, depressive symptoms based on the PHQ-2, and a positive response to any of the social needs questions. Interaction effects were also explored between the independent variables and age, sex, and race in separate models. Any significant interaction effects at *P*<.05 were included in the final models.

The diagnostic accuracy of the 3 “research perception” questions to predict positive response on the “research recruitment” question was assessed using receiver operating characteristic curve analysis. Area under the curve, the Youden index, sensitivity, specificity, negative predictive value, and positive predictive value were calculated for responses of “strongly agree” or “agree” as well as “very important” or “important” on the “research perception” questions. Statistical significance was established throughout at *P*<.05. All phase 2 analyses were conducted using SAS 9.4 (SAS Institute Inc).

## Results

### Phase 1: Qualitative Data Collection

The 32 participants who completed the cognitive interviews had a range of self-reported health conditions. Of the 32 participants, 13 (41%) were female, 13 (41%) were aged 65 years or older, and 11 (34%) had previously participated in a clinical research study (Table 1).

**Table 1:** Characteristics of participants in cognitive interviews (phase 1; n=32)^a^.

Characteristics			Participants, n (%)
**Age group (y)**			
	21-54	7 (22)	
	55-64	12 (37)	
	≥65	13 (41)	
Sex: female			13 (41)
Participated in clinical research study at Cleveland Clinic			11 (34)
**Educational level**			
	High school graduate	3 (9)	
	Some college or graduated 2-year college	9 (28)	
	Graduated 4-year college	8 (25)	
	Postgraduate degree	12 (37)	
**Race**			
	Asian	2 (6)	
	Black	6 (19)	
	White	22 (69)	
	Other	2 (6)	
**Marital status**			
	Married or partnered	22 (69)	
	Single	4 (12)	
	Divorced or widowed	6 (19)	
**Self-reported health condition**			
	Excellent	8 (25)	
	Good	8 (25)	
	Fair	11 (34)	
	Poor	5 (16)	

^a^Cognitive interviews were performed between October 31, 2017 and November 14, 2017 (for details, refer to the Cognitive Interviews subsection under Phase 1: Qualitative Study to Identify Optimal Question on Perception of Research) to identify a question that would best capture patients’ interest in participating in clinical research.

Data saturation was reached, with participants in the cognitive interviews voicing strong support for clinical research, recognizing its important role in advancing patient care advancement, and agreeing that it should be part of Cleveland Clinic’s mission (Table 2). Participants indicated that they preferred receiving information about research opportunities in person directly from their physicians, citing the trust-based relationship with their health care providers and their health care providers’ unique understanding of their medical needs. While participants also viewed emails, MyChart messages, and telephone calls as effective ways to learn about research eligibility, they wanted these to serve as secondary forms of communication after initial discussion with their physicians.

Participants held highly favorable views toward research, with 97% (31/32) agreeing or strongly agreeing that it should be part of the institution’s mission and 100% (32/32) affirming that research enhances patient care. Likewise, nearly all respondents indicated that they would consider participating in research if it benefited them (32/32, 100%) or others (31/32, 97%). Participants indicated that they feel it is important to help others, and many indicated feeling a sense of responsibility to give back; however, they would not participate in a clinical study to help others if they were physically or mentally harmed in the process. Of the 32 participants, 31 (97%) also expressed that it was important or very important to be informed about clinical research opportunities for which they were eligible. Furthermore, a few (2/32, 6%) said that they would be disappointed if their physician did not inform them about clinical studies for which they may be eligible candidates.

There was slightly less consensus on the significance of practical factors such as location, time commitment, and compensation (Table 3).

Participants found all questions to be clear and easy to understand, with no suggestions for additional questions. As the most effective question for gauging patients’ interest in research participation, they identified the following: “I would consider participating in a clinical research study if it could potentially help me.” This question, along with 2 other candidates, was chosen for piloting in clinical practice. The selection of these questions was based on participant feedback, the distribution of participant responses, and their perceived relevance. The additional 2 questions were the following: “I would consider participating in a clinical research study if it could potentially help others” and “How important do you feel it is for your healthcare provider to let you know of research trials for which you may be eligible?”

**Table 2:** Candidate “research perception” questions assessed in cognitive interviews and participant responses (phase 1; n=32)^a^. Participants responded to the following item: “Select the most appropriate response for each of the following questions.”

Questions	Strongly agree, n (%)	Agree, n (%)	Disagree, n (%)	Strongly disagree, n (%)
I feel that research should be part of Cleveland Clinic’s mission	24 (75)	7 (22)	1 (3)	0 (0)
I feel that research is important to be able to improve patient care	28 (88)	4 (12)	0 (0)	0 (0)
I would consider participating in a clinical research study if it could potentially help me^b^	23 (72)	9 (28)	0 (0)	0 (0)
I would consider participating in a clinical research study if it could potentially help others^b^	21 (66)	10 (31)	0 (0)	1 (3)

^a^Cognitive interviews were conducted between October 31, 2017 and November 14, 2017 (for details, refer to the Cognitive Interviews subsection under Phase 1: Qualitative Study to Identify Optimal Question on Perception of Research).

^b^These questions were selected for pilot implementation in electronic questionnaires.

**Table 3:** Candidate “research perception” questions assessed in cognitive interviews and participant responses (phase 1; n=32)^a^. Participants responded to the following question: “How important would each of the following factors be in your decision to participate as a volunteer in a clinical research study?”

Questions	Very important, n (%)	Somewhat important, n (%)	Not very important, n (%)	Not at all important, n (%)
Level of personal risk for adverse health outcomes	20 (63)	9 (28)	2 (6)	1 (3)
The location of the clinical research study is easily accessible	13 (50)	7 (22)	2 (6)	7 (22)
Whether you would be paid to participate	1 (3)	10 (31)	8 (25)	13 (41)
The amount of time commitment to participate	12 (41)	8 (28)	3 (10)	6 (21)
How important do you feel it is for your healthcare provider to let you know of research trials for which you may be eligible?^b^	22 (69)	9 (28)	0 (0)	1 (3)

^a^Cognitive interviews were performed between October 31, 2017 and November 14, 2017 (for details, refer to the Cognitive Interviews subsection under Phase 1: Qualitative Study to Identify Optimal Question on Perception of Research).

^b^This question was selected for pilot implementation in electronic questionnaires.

### Phase 2: Cross-Sectional Cohort Study

A total of 1077 patients completed the “research recruitment” question and at least 1 of the 3 “research perception” questions and were included in the study cohort (mean age 48.3, SD 16.3 years; 625/1065 female, 58.68%; 661/1005 White, 65.77%; Table 4). Mean PROMIS GH physical and mental health summary scores were 49.4, SD 8.8 and 49.6, SD 9.6, respectively, similar to the mean of the US general population. At least moderate depressive symptoms were indicated by 11% (106/936) of the patients. Moreover, 14.1% (148/1050) indicated that they had concerns about paying their bills, and 2.57% (27/1051) indicated that they had difficulty with transportation to their medical appointments.

**Table 4:** Characteristics of patients completing electronic questions stratified by interest in a precision medicine registry (phase 2)^a^.

Characteristics				All patients completing questions		Patients who did not wish to be contacted about the precision medicine registry		Patients who wished to be contacted about the precision medicine registry		*P* value	
Age (y), mean (SD)				48.3 (16.3)		47.2 (16.4)		51.4 (15.6)		<.001	
Female, n (%)				625/1065 (58.7)		457/789 (57.9)		168/276 (60.9)		.35	
**Race, n (%)**											.96
	Black		254/1005 (25.3)		191/750 (25.5)		63/255 (24.7)				
	White		661/1005 (65.8)		491/750 (65.5)		170/255 (66.7)				
	Other		90/1005 (9)		67/750 (8.9)		23/255 (9)				
Married, n (%)				530/1047 (50.6)		394/780 (50.5)		136/267 (50.9)		.90	
Household income (in units of US $10,000), mean (SD)				4.80 (2.04)		4.81 (2.02)		4.80 (21.1)		.94	
**Patient-reported outcomes, mean (SD)**											
	**PROMIS GH^b^**										
		Physical health	49.4 (8.8)		50.4 (8.3)		46.7 (9.4)		<.001		
		Mental health	49.6 (9.6)		50.4 (9.4)		47.3 (9.9)		<.001		
	**PHQ-2^c^ depression screen**		0.78 (1.34)		0.68 (1.27)		1.05 (1.51)		<.001		
		Depressed mood^d^, n (%)	106/963 (11)		68/711 (9.6)		38/252 (15.1)		.02		
	**Social needs questions, n (%)**										
		Payment trouble	148/1050 (14.1)		84/777 (10.8)		64/273 (23.4)		<.001		
		Transportation trouble	27/1051 (2.6)		11/778 (1.4)		16/273 (5.9)		<.001		
		Food trouble	431050 (4.1)		19/777 (2.4)		24/273 (8.8)		<.001		
		Housing trouble	28/1050 (2.7)		14/777 (1.8)		14/273 (5.1)		.003		
		Any social needs	166/1050 (15.8)		94/777 (12.1)		72/273 (26.4)		<.001		

^a^Patients seen in primary care clinics completed these electronic patient-reported outcomes as part of the standard of care.

^b^PROMIS GH: Patient-Reported Outcomes Measurement Information System Global Health.

^c^PHQ-2: Patient Health Questionnaire-2.

^d^Depressed mood based on PHQ-2 score of ≥3.

Of the 1077 patients, 278 (25.8%) responded affirmatively to the “research recruitment” question, indicating that they were interested in being contacted about the precision medicine registry. Patients who responded affirmatively were older than those who responded no (mean 51.4, SD 15.6 years vs mean 47.2, SD 16.4 years), had worse self-reported physical and mental health conditions, were more likely to report depressive symptoms (38/252, 15.1% vs 68/708, 9.6%), and indicated more social needs (72/273, 26.4% vs 94/777, 12.1%).

**Figure 2:**
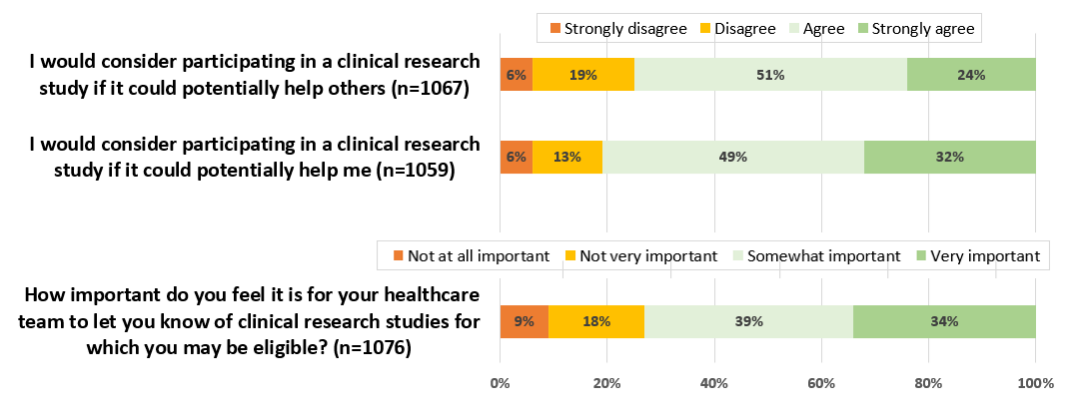
Responses to “research perception” questions (phase 2 cohort study).

The majority of the patients responded positively to the “research perception” questions: 75% (800/1067) agreed or strongly agreed that they would consider participating in a clinical research study if it could potentially help others, while 81.02% (858/1059) indicated that they would consider participating if it would help them (Figure 2). In addition, 72.96% (785/1076) of the respondents felt that it was important for their health care team to let them know of clinical research studies for which they may be eligible.

More positive responses to all 3 “research perception” questions were significantly associated with interest in the precision medicine registry. A dose-response relationship was demonstrated for the responses to each question (Table 5). For the question “I would consider participating in a clinical research study if it could potentially help others,” responses of “agree” and “strongly agree” were significantly associated with interest in the precision medicine registry (adjusted odds ratio 8.40, 95% CI 2.48-28.5 and adjusted odds ratio 17.6, 95% CI 5.08-61.1, respectively). The multivariable models incorporating demographic and health factors alongside “research perception” questions demonstrated superior discriminative ability compared to models using “research perception” questions alone, with C-statistic values ranging from 0.716 to 0.752 in adjusted models compared to values ranging from 0.628 to 0.650 in unadjusted models (Table 5). The highest discrimination was achieved by the model that included the “research perception” question “I would consider participating in a clinical research study if it could potentially help me” (C-statistic=0.752).

**Table 5:** Multivariable logistic regression models predicting interest in a precision medicine registry based on “research perception” questions (phase 2)^a^.

Covariates and response options			Adjusted odds ratio (95% CI)		*P* value		C-statistic	
“**I would consider participating** **in a clinical research study if it could potentially help others”** **(reference: “strongly disagree”)**								0.746
	Disagree	2.39 (0.66-8.71)		.19				
	Agree	8.40 (2.48-28.5)		<.001				
	Strongly agree	17.6 (5.08-61.1)		<.001				
“**I would consider participating in a clinical research study if it could potentially help me”**^b^ **(reference: “strongly disagree”)**								0.752
	Disagree	1.14 (0.29-4.55)		.85				
	Agree	5.50 (1.63-18.5)		.006				
	Strongly agree	11.1 (3.28-37.8)		<.001				
“**How important do you feel it is for your healthcare provider to let you know of research trials for which you may be eligible?” (reference: “not at all important”)**								0.716
	Not very important	2.23 (0.91-5.48)		.28				
	Somewhat important	2.91 (1.26-6.72)		.01				
	Very important	6.36 (2.77-14.6)		<.001				

^a^The independent variables in these models were the “reception perception” questions, and the dependent variable was interest in the precision medicine research registry. The models adjusted for age, sex, race, marital status, median household income, depressive symptoms, Patient-Reported Outcomes Measurement Information System Global Health, any social needs, and significant interactions.

^b^Significant interaction with current age; estimates presented for the model without the interaction effect.

The “research perception” questions demonstrated high sensitivity but limited specificity (Table 6). For responses of “agree” or “strongly agree” or “important” or “very important,” sensitivity exceeded 85% across all questions. Similarly high negative predictive values (>85%) indicated that patients who responded negatively to these questions were unlikely to express interest in the precision medicine registry. However, the questions demonstrated low specificity (24%-31%) and positive predictive values (30%), indicating that positive responses were not strong predictors of actual interest in the precision study.

**Table 6:** Unadjusted diagnostic accuracy of primary care patients’ responses to the 3 “research perception” questions in predicting patients’ interest in learning more about the precision medicine registry (phase 2).

Variables	“Research perception” questions		
	“I would consider participating in a clinical research study if it could potentially help others”: “strongly agree” or “agree”	“I would consider participating in a clinical research study if it could potentially help me”: “strongly agree” or “agree”	“How important do you feel it is for your healthcare provider to let you know of research trials for which you may be eligible?”: “very important” or “important”
AUC^a^	0.628	0.650	0.636
Youden index	0.183	0.186	0.158
Sensitivity (%)	88.4	94.6	84.9
Specificity (%)	30	24	31
PPV^b^ (%)	30.7	30.3	30
NPV^c^ (%)	88	92.7	85.5

^a^AUC: area under the curve.

^b^PPV: positive predictive value.

^c^NPV: negative predictive value.

## Discussion

### Principal Findings

This study demonstrated that the response to 1 question added to electronic patient questionnaire sets about patient perceptions regarding research may help prioritize recruitment efforts to patients who are more likely to participate in clinical research studies.

There are several potential approaches to using patient-entered data to aid in research recruitment (Table 7), with each having distinct advantages and limitations. While methods that directly assess patient eligibility and interest by using screening questions can reduce the research team’s workload, practical constraints within clinical care settings often limit their feasibility and may compromise respect for patient preferences.

**Table 7:** Options for using patient-entered data to aid patient recruitment into clinical trials.

Options	Strengths	Limitations
1. Ask specific eligibility questions for each clinical trial	Most directly useful Does not require patient to indicate interest in research	Adds to patient burden from question completion Most resource-intensive option to build and maintain May require preceding patient consent to ask research eligibility questions or have the ability for patients to opt out
2. Ask common eligibility questions that can be used across the clinical trials for each area	Requires fewer resources to update than option 1 Does not require patients to indicate interest in research	Adds to patient burden from question completion Requires build of custom questions for each research area May require preceding patient consent to ask research eligibility questions or have the ability for patients to opt out
3. Ask about general interest in participating in, or being contacted about, research	Simpler to implement Can be used to prioritize patients to approach about specific trials	Soliciting research participation from patients who have explicitly declined interest represents a violation of their autonomy Patients who respond “not interested” to the patient-entered data question may still be interested upon discussion with their care providers Must assess preliminary study eligibility through another route
4. Ask general question about perception of research (treat as “patient attribute”)	Simpler to implement Can be used to prioritize patients to approach about specific trials	Efficacy of this approach requires empirical testing Must assess preliminary study eligibility through another route

Capturing patients’ attitudes toward research offers a balanced solution that preserves autonomy and prevents the frustration that may occur when those who have explicitly declined contact about research are nonetheless approached. In addition, this approach is concise, straightforward to implement, and requires minimal ongoing maintenance.

The question “I would consider participating in a clinical research study if it could potentially help me” demonstrated superior performance metrics, with both a higher area under the curve and greater sensitivity compared to the alternative questions. However, its direct personal nature may make it less suitable for integration into general clinical questionnaires. Conversely, the question “How important do you feel it is for your healthcare provider to let you know of research trials for which you may be eligible” had the lowest C-statistic value and sensitivity but may have a better contextual fit within standard previsit questionnaires, potentially making it more practical for routine clinical implementation. Specificity was relatively low for all questions; in this context, optimizing sensitivity over specificity can be considered advantageous, as it minimizes the risk of missing potentially interested participants while accepting a higher rate of false positives.

The cognitive interviews revealed that patients prefer to hear about research opportunities directly from their physician. A preference for health care provider to patient discussion about research has been noted by others [24-26]. Incorporating “research perception” questions within the clinical workflow supports this preference by enabling physicians to prioritize recruitment efforts during their office visit based on patient responses indicating an interest in research. This approach is used by the Movement Disorder Section of the Neurological Institute at our health system, where patients answer the “research perception” question “I think it’s important to be told of research trials for which I may be eligible” alongside other clinical questions before their visit. In the experience of the authors, this screening method has proven effective in prompting clinicians to spend additional time discussing relevant studies with patients who express interest.

Our adjusted models, which contained a “research perception” question and additional variables, had improved discriminative capacity. If electronically implemented, they could allow a more precise estimation of patients’ interest in research. However, targeting participants based on demographic and health status characteristics could introduce greater patient selection bias, reducing a study’s generalizability. Adjusted models would also be more resource intensive to implement.

Recent advances in EHR systems, apart from patient-entered data functionality, have also streamlined the patient recruitment process for clinical research. Many now have query capabilities that enable research teams to efficiently identify potentially eligible patients by searching structured data fields for specific eligibility criteria [27]. This automation has reduced the need for developing custom patient questionnaires to screen patients for eligibility. Artificial intelligence applied to EHR records can also now screen patients for eligibility criteria [28,29]. In addition, EHR systems can now directly engage potential participants through patient portals, automatically notifying them of trials that match their clinical profile. Although only a small percentage of patients contacted this way enroll as participants, this approach is effective at increasing recruitment because of the large volume of patients contacted through patient portal messages [30-32]. Integrating this EHR-based recruitment method with the patient attribute question can create a synergistic approach to optimize patient enrollment. Using multiple recruitment methods can increase enrollment yield [33,34].

Although this study was conducted at a single institution, the proposed approach is readily adaptable to other health systems that routinely collect patient-reported data during clinical visits. As technology advances, patient-reported data collection is becoming increasingly commonplace [35]. Adding a single question that requires no score calculations to existing questionnaire sets is straightforward and minimally increases the burden of patient response.

### Limitations

Several important limitations should be noted. First, although participants in the cognitive interviews reported a wide range of health conditions, they had more education than the general population, and approximately one-third of the interviewees (11/32, 34%) had previously participated in a clinical research study. Their opinions may differ from those with different educational backgrounds and experience with clinical research. Second, our assessment focused solely on the relationship between “research perception” questions and interest in a precision medicine registry. Patient attitudes may vary substantially across different types of clinical research. Third, responses to research participation questions might differ significantly if patients were facing an actual serious medical diagnosis rather than a hypothetical scenario. Fourth, our methodology for maintaining screening logs limited our ability to track actual study participation in the precision medicine registry. Instead, we could only measure the relationship between “research perception” questions and patients’ initial interest in learning more about the study. Finally, relying on patient-entered questionnaires as a screening tool has inherent limitations. This approach would only capture data from patients who complete electronic questionnaires, potentially underrepresenting racial and ethnic minority groups or populations considered vulnerable who may have limited access to patient portals [36].

### Conclusions

The ability to identify patients who have a greater likelihood of participating in research studies by assessing patients’ attitudes toward research is a helpful tool to aid patient recruitment into clinical research. This approach allows research teams to prioritize their recruitment efforts more effectively without relying on strict eligibility criteria. It provides a framework for more nuanced and respectful engagement in the recruitment process, ultimately serving both research efficiency and patient experience. The use of broad indicators rather than specific eligibility questions creates a more flexible and sustainable recruitment strategy that respects patient preferences while maximizing research participation opportunities. Further evaluation of this approach is warranted to assess the ability to use patient-reported questions on attitudes toward research for other types of clinical trials that vary by risk of adverse outcomes, level of patient burden, and health condition.

## Appendix

Multimedia Appendix 1 Interview guide.

Multimedia Appendix 2 Precision medicine registry recruitment questionnaire.

## Abbreviations

EHR: electronic health record
PHQ-2: Patient Health Questionnaire-2
PROMIS GH: Patient-Reported Outcomes Measurement Information System Global Health

## Data Availability

The datasets generated and analyzed during this study are available from the corresponding author on reasonable request.
