# Supplementary material for: Improving Recruitment Into Research Studies via Electronically Collected Patient-Entered Data: Mixed Methods Study
Source: J Med Internet Res. 2025 Oct 29;27:e77720. doi: 10.2196/77720 (PMC12612641; doi:10.2196/77720)
Supplement: Multimedia Appendix 1 [file jmir_v27i1e77720_app1.doc]

**AId TO Research Recruitment (AvIaTOR): a mixed methods study to improve recruitment into research studies via electronically collected patient-entered data**

**Multimedia Appendix 1**

**Interview Guide:**

**Aviator—Patient Perception of Clinical Research**

**Participant Name: _________________ Date of Interview: ___________________**

**Introduction**

Thank you for agreeing to participate in this interview! The Cleveland Clinic **Center for Clinical Genomics** is sponsoring this study. The main purpose of our discussion today is to get ***specific feedback*** from you so that Cleveland Clinic can enhance the effectiveness of patient recruitment into clinical research studies performed within the Cleveland Clinic Health System.

All results of this study will be reported in the aggregate—I am conducting a total of 32 patient telephone interviews and specific names **will not** be used in this report. This is a free-flowing discussion and there are no right or wrong answers. To thank you for your time, you will receive a **$25 Amazon gift code** within 24 hours after this call.

- Disclose being recorded so that an accurate summary report can be written
- Disclose who on the call
- Want honest feedback—anything you say will be kept confidential

**Interest in Clinic Research at Cleveland Clinic**

| **1.** What does clinical research mean to you? |  |
| --- | --- |
| **2.** How interested are you in clinical research?  (Very Interested, Somewhat Interested, Not very interested, Not at all Interested) |  |
| **2a. [If Somewhat or Very Interested]**  What peaks your interest in clinical research? |  |
| **2b. [If Not Very Interested or Not at all Interested]**  Tell me what makes you feel that way? |  |
| **3.** Have you ever participated in a clinical research study at Cleveland Clinic?  If Yes: Can you tell me a little bit about your experience?   - How did you hear about the study? - Describe your process of deciding whether or not to participate in the study? - How was your experience in the study? |  |
| **4.** Has a loved one ever participated in a clinical research study at Cleveland Clinic?  If Yes: Can you tell me a little bit about that experience?   - What did you think about your loved one participating in the research study? |  |

**Statements about Clinical Research**

| **5.** Now I want to know how much you agree or disagree with the following statements about clinical research.  (Strongly Agree, Agree, Disagree, Strongly Disagree)  **“I feel that research should be a part of Cleveland Clinic’s mission.”**   - Probe: What makes you [insert answer] that research should be a part of Cleveland Clinic’s mission? - Probe: Is there anything about this question that could be confusing to patients? |  |
| --- | --- |
| **6.** (Strongly Agree, Agree, Disagree, Strongly Disagree)  **“I feel that research is important to be able to improve patient care.”**   - Probe: What makes you [insert answer] that research is important to be able to improve patient care? - Probe: What does “improving patient care” mean to you? - Probe: Is there anything about this question that could be confusing to patients? |  |
| **7.** (Strongly Agree, Agree, Disagree, Strongly Disagree)  **“I would consider participating in a clinical research study should it potentially help me.”**   - Probe: What makes you [insert answer] that you would consider participating in a clinical research study should it potentially help you? - Probe: You said you ___ that you would consider participating in a clinical research study should it potentially help you. What does that mean to you? - Probe: How would you determine if it could potentially help you? - Probe: What specific instances would you consider participating in a clinical research study if it would help you? - Probe: Is there anything about this question that could be confusing to patients? |  |
| **8.** (Strongly Agree, Agree, Disagree, Strongly Disagree)  **“I would consider participating in a clinical research study if it could potentially help others.”**   - Probe: What makes you [insert answer] that you would consider participating in a clinical research study if it could potentially help others? - Probe: You said you ___ that you would consider participating in a clinical research study if it could potentially help others. What does that mean to you? - Probe: How would you determine if it could potentially help others? - Probe: What specific instances would you consider participating in a clinical research study if it would help others? - Probe: Is there anything about this question that could be confusing to patients? |  |

**Decision to Participate in Clinical Research Studies at Cleveland Clinic**

| **9.** How important would each of the following factors be in your decision to participate as a volunteer in a clinical research study at Cleveland Clinic?  (Very Important, Somewhat Important, Not Very Important, Not at All Important)   - Low personal risk for adverse health outcomes - The location of the clinical research study is easily accessible - Whether you would be paid to participate - **The amount of time commitment to participate** |  |
| --- | --- |
| **10.** Would you be more or less likely to participate in a clinical research study if it involved analysis of your DNA?  Example (if needed) An example of a DNA analysis is identifying genes associated with cancer or medical conditions such as high blood pressure  (Much More Likely, More Likely, Less Likely, Much Less Likely)  Probe: What Makes you feel that way? |  |
| **11.** How important do you feel it is for your healthcare provider to let you know of research trials for which ***you*** may be eligible?  (Very important, Somewhat important, Not very important, Not at all important)  Probe: What Makes you feel that way? |  |
| **11a. Which of the above questions do you feel are more likely to capture a patient’s interest in participating in a clinical research study?** |  |

**Conclusion**

| **12.** Based on today’s discussion, what  **one thing would you most recommend**  to Cleveland Clinic in order to help  recruit the appropriate patients into participating in  clinical research studies? |  |
| --- | --- |
| **13..** These are all of the questions that I have for you today/tonight; however I would like to know if you have anything else you would like to share about your perceptions of clinical research at Cleveland Clinic? |  |
| **14.** Thank you so much for your time and opinions! As a thank you for sharing your opinions with me today, you will receive a $25 Amazon gift code via email within the next 24 hours. What **email address** would you like me to send this gift code to? | **Email Address: _________________** |
