# Supplementary material for: Improving Recruitment Into Research Studies via Electronically Collected Patient-Entered Data: Mixed Methods Study
Source: J Med Internet Res. 2025 Oct 29;27:e77720. doi: 10.2196/77720 (PMC12612641; doi:10.2196/77720)
Supplement: Multimedia Appendix 2 [file jmir_v27i1e77720_app2.docx]

**AId TO Research Recruitment (AvIaTOR): a mixed methods study to improve recruitment into research studies via electronically collected patient-entered data**

**Multimedia Appendix 2**

**Precision Medicine Recruitment Questionnaire**

*Precision Medicine is defined as customized healthcare with the focus of treating each patient through an individual focus which takes into account individual differences in genes, environment, and lifestyle. Cleveland Clinic will be actively enrolling participants in a Precision Medicine Registry. The registry is a list of individuals, both adults and children, who are interested in learning more about their health in a personalized fashion, so that researchers can contact them about future available research studies. Agreeing to have your name and contact information placed in the Precision Medicine Registry does not enroll you into future research studies. This will allow researchers to contact you and present studies for your consideration in the future.*

**Would you or your family like to be contacted by Cleveland Clinic team to learn more? (Only asked once)**

- Yes
- No (skip to the end of the questionnaire)

**What is your preferred method of contact?**

- Text (United States only; standard text rates apply)
- Phone call (United States only)
- Email
- US Mail
